# Melanoma differentiation-associated gene 5 amyopathic dermatomyositis following an acute *Mycoplasma pneumoniae* infection: a case report

**DOI:** 10.1186/s13256-022-03616-z

**Published:** 2022-11-01

**Authors:** Jessica Hoey, Jenny Lue Solomon, Brandon Kim, Steven Carsons, Julie Nusbaum

**Affiliations:** 1 Division of Rheumatology, Allergy, and Immunology, NYU Long Island School of Medicine, Mineola, NY USA; 2 Department of Internal Medicine, NYU Langone Hospital, Long Island, Mineola, NY USA

**Keywords:** Case report, MDA5 amyopathic dermatomyositis, *Mycoplasma pneumoniae*, Myositis, Dermatomyositis, Interstitial lung disease, Arthritis, Palmar papules, Oral ulcers

## Abstract

**Background:**

A previously healthy young male of Southeast Asian descent presented with 6 weeks of fevers, cough, mucocutaneous ulcers, arthritis, and myalgias. Initial workup revealed positive *Mycoplasma pneumoniae* immunoglobulin M, and the patient was treated with antibiotics without relief of symptoms. Rheumatologic workup revealed highly positive melanoma differentiation-associated gene 5 antibody. Viral infections are thought to potentially trigger loss of self tolerance, and prompt the autoimmunity cascade that can result in conditions such as dermatomyositis. To our knowledge, this is the first case report demonstrating a non-viral infection, specifically *Mycoplasma pneumoniae*, as the inciting infectious trigger for the anti-melanoma differentiation-associated gene 5 dermatomyositis subtype.

**Case presentation:**

A 20-year-old southeast Asian–American male with no significant past medical history presented with symptoms of intermittent fevers, nonproductive cough, dry eyes, oral ulcers, rash, arthritis, and myalgias. The patient was noted to have erythematous papules across the bilateral hands along the lateral digits and palms, as well as synovitis involving the bilateral hands and feet. Immunoglobulin M antibodies were positive for *Mycoplasma pneumoniae*. The patient was diagnosed with mycoplasma pneumonia. The patient did not respond to a course of antibiotics, leading to rheumatological testing that found highly positive melanoma differentiation-associated gene 5 autoantibody. Muscle enzyme and electromyography testing were normal, indicating clinically amyopathic disease. Methylprednisolone was initiated, with resolution of fevers and improvement of arthritis and myalgias. The cutaneous lesions on the digits and palms improved.

**Conclusions:**

This patient presented with symptoms of fever, cough, oral ulcers, rashes, and arthritis, and blood work demonstrated the presence of immunoglobulin M antibodies to *Mycoplasma pneumoniae*. Despite antibiotic treatment for the presumed diagnosis of *Mycoplasma pneumoniae* infection, the patient did not improve, prompting rheumatological workup and revealing melanoma differentiation-associated gene 5 autoantibodies. This case suggests that infections, other than viral, can trigger the autoinflammatory cascade, leading to the development of amyopathic melanoma differentiation-associated gene 5 dermatomyositis.

## Background

Anti-melanoma differentiation-associated gene 5 (MDA5) dermatomyositis has been described in the literature over the last decade. The antibody, originally designated anti-clinically amyopathic dermatomyositis-140 (CADM-140), is a ribonucleic acid (RNA)-specific helicase involved in antiviral immune responses. MDA5 behaves as a pattern recognition receptor for intracellular viral RNA, leading to the suppression of viral particle replication [1, 2]. It has been hypothesized that viral infections might be a trigger for the loss of self tolerance and initiate a cascade of autoimmunity that may lead to certain autoimmune conditions, including dermatomyositis [3].

Our patient had positive immunoglobulin M (IgM) titers for *Mycoplasma pneumoniae* and negative immunoglobulin G (IgG). Intracellular microorganisms, such as *M. pneumoniae*, are believed to activate B and T lymphocytes via molecular mimicry crucial for the development of autoimmune diseases [7]. *M. pneumoniae* has been reported as the trigger of juvenile dermatomyositis in a 14-year-old girl [8]. To our knowledge, this is the first case report demonstrating *M. pneumoniae* as the inciting infectious trigger specifically for the MDA5 dermatomyositis subtype with clinically amyopathic disease (CADM).

## Case presentation

A previously healthy 20-year-old southeast Asian–American male presented with 6 weeks of intermittent fevers, nonproductive cough, dry eyes, oral ulcers, rashes, arthritis, and myalgias. The patient’s vitals were temperature of 102°F, heart rate 99 beats per minute, respiratory rate 22 breaths per minute, blood pressure 112/63 mmHg, and oxygen saturation 96% on room air. On physical examination, the patient was not in acute distress, no scleral icterus was noted, extraocular movements were intact, with oral ulcers on the hard palate. The patient did not have evidence of jugular vein distention (JVD), carotid bruits, and no palpable cervical or axillary lymphadenopathy. Cardiac examination found normal rate and rhythm with audible S1 and S2. Lung examination was clear to auscultation bilaterally without rales, rhonchi, or wheezes. The patient’s abdomen was soft, nontender, and nondistended with normoactive bowel sounds. The patient noted papules along lateral digits and tender palmar maculopapular lesions, synovitis of the hands and feet, but no noticeable edema of all extremities. Neurological examination was without focal deficits, and he was alert, awake, and oriented, answering questions appropriately, with strength of 5/5 on upper and lower extremities. The patient did not have significant past medical history, did not take any medications, and his only surgery was a circumcision. The patient’s family history was negative for rheumatological disease, his mother is a thalassemia carrier. The patient denies any tobacco, alcohol, or drug use. The patient is a university student.

**Fig. 1:**
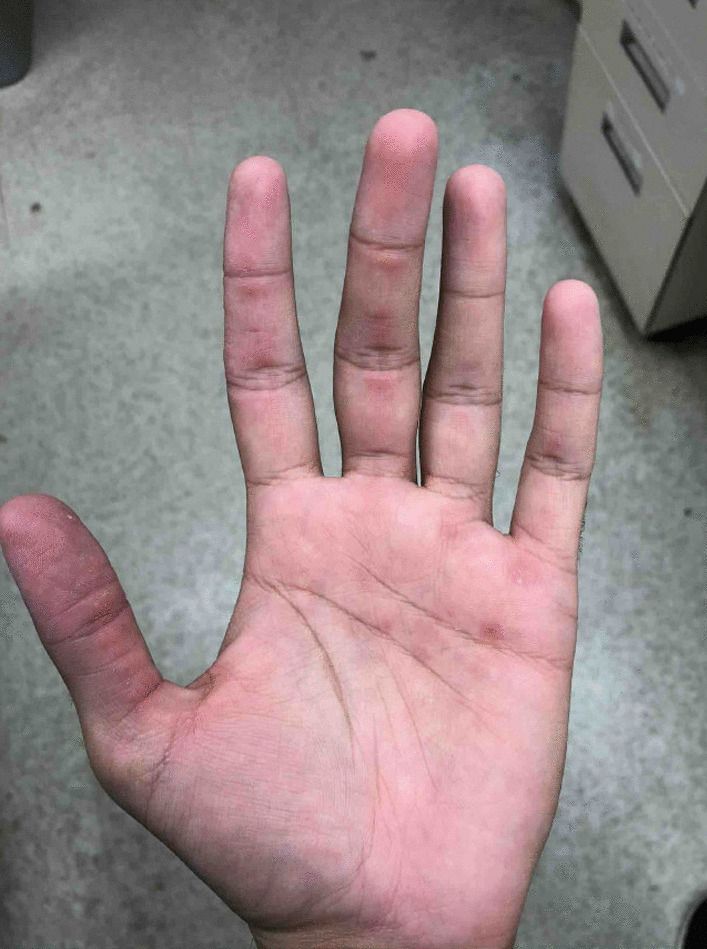
The patient presented with tender palmar

Three days prior to admission, the patient sought his primary care physician who prescribed 5 days of azithromycin 500 mg on day 1, then 250 mg on days 1–5, and a methylprednisolone dose pack. An outpatient chest computed tomography (CT) without contrast showed an ill-defined density in the right lower lobe and scattered micronodules. The patient did not improve and presented to the hospital (Fig. 1).

**Fig. 2:**
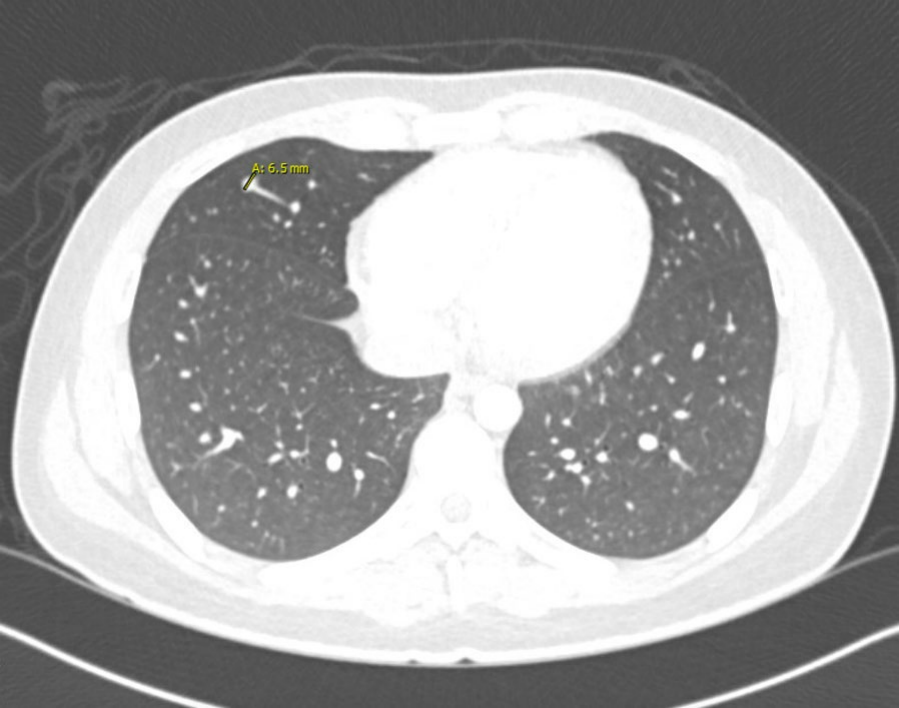
Chest CT imaging showing a 6.5mm right middle lobe nodulemaculopapular lesions

**Fig. 3:**
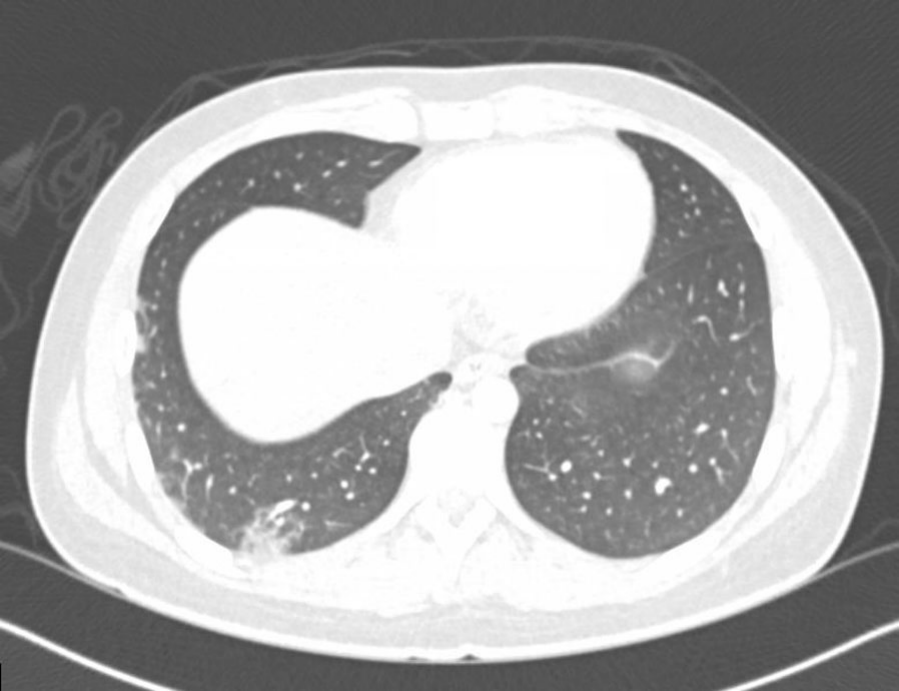
Chest CT imaging showing ground glass opacities

**Fig. 4:**
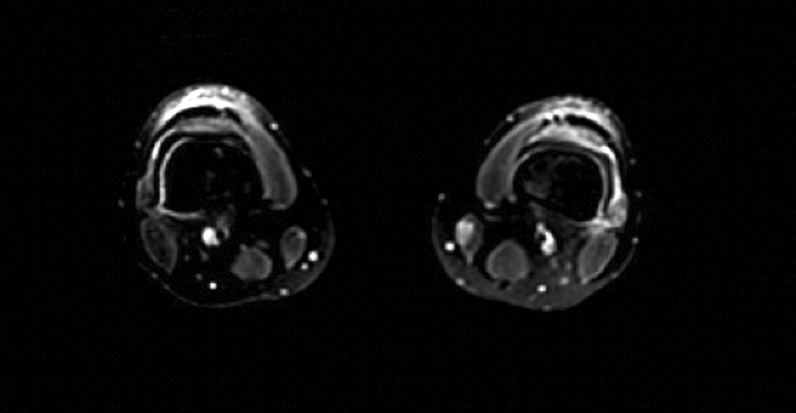
MRI of lower extremities showing nonspecific edema of left distal sartorius muscle

On hospital day 1, X-rays of hands and wrists were normal. Repeat CT imaging revealed additional ground glass opacities in the periphery of the right lung and a 6.5 mm right middle lobe nodule (Figs. 2 and 3). MRI of the lower extremities (Fig 4) showed nonspecific edema of the left distal sartorius muscle, but this finding was deemed to be nonspecific and not relevant to the patient’s condition when reviewed by the musculoskeletal radiologists and the treating physicians. There was an absence of other evidence to suggest the presence of muscle inflammation. Echocardiogram and CT and MRI imaging of brain, sinuses, and spine were normal. A skin biopsy of the left palm revealed superficial mounds of parakeratosis and sparse lymphocytic inflammation, suggestive of a resolving spongiotic or psoriasiform dermatitis. Electromyography and nerve conduction studies were normal. Laboratory tests were as follows: hemoglobin 11.4 g/dL (13.7–17.5 g/dL), absolute lymphocytes 1000/µL (1.3–3.6 1000/µL), creatinine 0.7 mg/dL, alanine transaminase (ALT) 91 IU/L (0–50 IU/L), aspartate aminotransferase (AST) 58 IU/L (5–34 IU/L), ferritin 920 ng/mL (12–300 ng/mL), and creatine phosphokinase (CPK) 91 IU/L (55–170 IU/L). Urinalysis was positive for trace ketones, otherwise negative for leukocyte esterase/nitrites/blood/protein. Infectious workup revealed positive *M. pneumoniae* IgM and negative IgG, Epstein–Barr viral capsid antigen (VCA) IgM, and parvovirus B19 IgM. Epstein–Barr virus (EBV) and parvovirus B19 PCR were negative. Severe acute respiratory syndrome coronavirus 2 by nucleic acid amplification test (SARS-CoV-2 by NAAT) was negative. Two sets of anaerobic and aerobic blood cultures drawn from two separate venipuncture sites transported at room temperature had no plated growth after 5 days. No cultures for fungi were collected. Urine culture grew < 1000 colony forming units per milliliter. The patient completed 10 days of oral doxycycline 100 mg every 12 hours for *M. pneumoniae* infection without improvement. Notably, *M. pneumoniae* IgG subsequently turned positive.

The patient continued to have fevers, oral ulcers, arthritis, and myalgias. An antinuclear antibody (ANA) titer 1:160 with homogeneous immunofluorescence pattern and weakly positive Sjogren’s syndrome-related antigen A (SSA)/anti-Ro antibody were noted. A highly positive anti-MDA5 was measured by qualitative immunoprecipitation assay on hospital day 15 and confirmed on repeat testing.

Intravenous methylprednisolone 60 mg daily was initiated for 2 days, with resolution of fevers and improvement of arthritis and myalgias. The patient was tapered to oral prednisone 30 mg twice daily, with improvement of cutaneous lesions on the digits and palms. He was referred to pulmonology for close monitoring with pulmonary function tests and serial CT chest imaging. Chest CT at 3 and 12 months after diagnosis showed no evidence of interstitial lung disease. The patient started treatment with azathioprine 50 mg daily as an outpatient; however, due to persistent transaminitis, was switched to mycophenolate mofetil initially on 500 mg twice daily and titrated up to 1000 mg twice daily. Owing to the side effect of diarrhea, he was then changed to mycophenolic acid 720 mg twice daily, which was well tolerated.

## Discussion and conclusions

The identification of mycoplasma as an inciting factor in the development of MDA5 dermatomyositis is novel and has not been well established. Our patient’s initial symptoms of mucocutaneous ulcers, erythematous papules on bilateral hands, fevers, cough, arthritis, and myalgias and the positive *M. pneumoniae* IgM, taken together with the subsequent positive anti-MDA5 antibody and improvement in symptoms upon initiation of steroids ultimately makes a strong case supporting the correct diagnosis of mycoplasma infection at presentation, and the subsequent diagnosis of MDA5 dermatomyositis. Other infectious or rheumatologic causes of the patient’s symptom complex were comprehensively ruled out. MDA5 dermatomyositis diagnosis was established with fulfillment of serologic and clinical criteria. Viral infections have been posited as the trigger for loss of self tolerance that incites an autoimmunity cascade that can cause dermatomyositis; however, in this case, uniquely, a nonviral infection likely triggered the anti-MDA5 dermatomyositis. The temporal relationship between the acute *M. pneumoniae* infection and MDA5 dermatomyositis suggests that the bacteria triggered the dermatomyositis.

Although there are no official diagnostic criteria, amyopathic dermatomyositis is often diagnosed by hallmark cutaneous manifestations, interstitial lung disease, and serologic testing positive for anti-MDA5 antibody, all in the absence of muscle involvement. Skin findings may include Gottron’s papules (violaceous papules on interphalangeal and metacarpophalangeal joints), Gottron’s sign (macular violaceous erythema over other joints), heliotrope eruption (violaceous erythema of the periorbital skin), and pink-violaceous erythema of scalp, neck, shoulders, extensor surfaces of upper extremities, upper back, and upper chest [2]. There are no well-established treatments of anti-MDA5 dermatomyositis, however, immunosuppression with glucocorticoids is widely used, as in this case [2].

This case report was limited by the inability to assess quantitative MDA5 antibody levels. Monitoring the trend in these antibody levels over the course of treatment, although not standard in the care of myositis patients, would have been informative in understanding the resolution of the patient’s symptoms. Additionally, we can identify a strong association, but cannot establish causation, of *M. pneumoniae* triggering myositis.

MDA5 dermatomyositis is classically characterized by cutaneous and oral ulcerations, painful palmar papules, mechanic’s hands, arthritis, and interstitial lung disease (ILD) [1]. In our case, these findings were initially attributed to *M. pneumoniae*, but were in fact features of MDA5 dermatomyositis. These distinctive characteristics may develop as a consequence of a vasculopathy seen specifically in MDA5 dermatomyositis [3]. While not seen in our patient, the presence of cutaneous ulcerations is also a strong predictor for the development of interstitial lung disease [1]. It is important to assess for ILD at the time of diagnosis because MDA5-positive patients are 20 times more likely to develop rapidly progressive ILD (RP-ILD) than those who are MDA5 negative [1]. MDA5 antibody has been identified in 10–30% of adults with dermatomyositis, the majority with CADM, as in our case [1, 3]. Certain ethnic groups, including Asians, have a lower incidence of myositis [1]. Our patient, of southeast Asian descent, had myalgias with isolated unilateral sartorius edema, but normal muscle enzymes and electromyography testing.

In summary, our patient presented with fevers, nonproductive cough, oral ulcers, rashes, and arthritis, which were initially attributed to an acute infection of *M. pneumoniae*. After poor response to antibiotics, additional workup revealed MDA5 autoantibodies. The timing of the *M. pneumoniae* infection with the manifestation of symptoms of anti-MDA5 dermatomyositis implies that the bacterial infection promoted the loss of self tolerance previously seen in viral infections and dermatomyositis.

Anti-MDA5 dermatomyositis presents with a unique subset of clinical features, including oral ulcers, distinct skin lesions, often amyopathic disease, and a significant risk for ILD, especially RP-ILD. Our patient’s case suggests that, in addition to the known relationship between viral infections and autoimmune disease, mycoplasma infections may also trigger an inflammatory cascade leading to autoimmune disease, in this case, MDA5 dermatomyositis. This has implications for our understanding of the pathogenesis of this type of dermatomyositis, suggesting that molecular mimicry by intracellular microorganisms may play a role in the activation of B-cell and T-cell-driven immune cascades.

## Data Availability

Information used for this case report can be made available from the corresponding author upon request.

## Abbreviations

MDA5: Melanoma differentiation-associated gene 5
Anti-CADM-140: Anti-clinically amyopathic dermatomyositis-140
RNA: Ribonucleic acid
*M. pneumoniae*: *Mycoplasma pneumoniae*
EBV: Epstein–Barr virus
ALT: Alanine aminotransferase
AST: Aspartate aminotransferase
CPK: Creatine phosphokinase
PCR: Polymerase chain reaction
SARS-CoV-2 by NAAT: Severe acute respiratory syndrome coronavirus 2 by nucleic acid amplification test
ANA: Antinuclear antibody
Anti-SSA: Anti-Sjogren’s syndrome-related antigen A
ILD: Interstitial lung disease
RP-ILD: Rapidly progressive interstitial lung disease
